# A New Minimally Invasive Procedure for Muscle, Back, Neck Pain and Radiculopathy - The Myofascial Nerve Block

**DOI:** 10.25107/2474-1655-v7-id2233

**Published:** 2022-06-24

**Authors:** S Omoigui, A Fadare

**Affiliations:** Department of Inflammation and Pain Research, LA Pain Clinic, USA

## Abstract

This is the first description of a procedure targeting the myofascial tissues since Janet Travell’s description of myofascial trigger points in 1942. However unlike trigger point injections, this minimally invasive myofascial nerve injection is performed differently and targets the myofascial tissues, peripheral innervations, posterior spinal structures and spinal nerve roots at the same time. It is different from a trigger point injection that aims to block trigger points within a muscle. Prolonged standing or sitting, posture and other multiple factors can create recurrent injuries with attendant inflammation and episodically aggravate pain. Thus there is a need for a simple intervention technique that can be performed from the medical clinic to the battlefield to quickly relieve inflammation and prevent chronic pain.

## Introduction

This myofascial nerve injection is also the first procedure for spine pain since epidural pain injection was first described by Jean-Anasthase Sicard in Paris on April 20^th^, 1901 [1,2]. However, at about the same time, Fernand Cathelin also from Paris had been treating patients with epidural injections for some months already [3].

Incidentally, a few months after we published our novel short needle technique for paraspinal muscle block in February 2016 [4,5], the Erector Spinae (ESP) nerve block was described in a publication in September 2016, as a regional block for thoracic neuropathic pain [6–8]. The injection is performed by a single shot or with a catheter insertion for continuous infusion (5 ml to 14 ml per hour) [9,10]. The primary mechanism is thought to be a direct effect of local anesthetic *via* physical spread and diffusion to the erector spinae muscles as well as neural structures in the fascial plane deep to the erector spinae muscles and adjacent tissue compartments [11]. Satisfactory results have been obtained in the treatment of both acute pain and chronic pain, and in some cases the ESP has replaced the use of epidural injections.

Clear solution of medication injected under pressure from a 30 G 5/8 inch (15.6 mm) needles travel a distance of 4 cm to 6 cm. As described in Sota Omoigui Short Needle Technique [4], utilizing the physics principle of the law of conservation of mass, the increased velocity of the injectate is sufficient to traverse the erector spinae muscles to the vertebral lamina and block the posterior spinal structures and nerves at the level injected [4]. Compressing the soft tissue with one hand, and injecting 1 ml to 2 ml Lidocaine 2% at the two most painful levels on each side of the vertebra or spinous process, we have been able to achieve 60% to 100% of relief of axial and radicular pain within 5 min. Total dosage of Lidocaine must be below the toxic dose of 3 mg/kg without epinephrine.

Due to the small size and length of the needle, this technique can be performed not just by pain specialist but by a primary care physician, physician assistant, nurse practitioner or any medical service provider trained to do intramuscular injections. This myofascial nerve block can be performed with or without imaging guidance in a variety of settings from the operating room to the medical clinic to the battlefield.

This myofascial nerve block may be used in combination with an anti-inflammatory regimen, including oral, IV or IM Steroid, Ketorolac/Diclofenac, Opioids, Ketamine, Ketorolac, Magnesium Sulfate, Kineret, CGRP Blockers, Botulinum Toxin etc. These block or inhibit various inflammatory mediators including prostaglandins (steroid, ketorolac/diclofenac), IL-1 Beta (Kineret) NMDA receptors (magnesium sulfate, ketamine), Substance P (Botulinum Toxin) etc [12].

Low back and neck pain causes more disability than any other and accounts for the third highest health care spending after diabetes and ischemic heart disease [13].

Subsequent to tissue injury, the initial immune reaction generates an inflammatory milieu of chemical mediators that include prostaglandin, interleukin 1-alpha, interleukin 1-beta, interleukin-4, Interleukin-6 and interleukin-8 nitric oxide, tumor necrosis factor alpha, histamine and serotonin [14,15].

Following this injury, there is increased nerve traffic in the sensory neurons that travel to the spinal cord and stimulate the release of inflammatory protein Substance P. The presence of Substance P and other inflammatory proteins such as Calcitonin Gene-Related Peptide (CGRP) neurokinin A and vasoactive intestinal peptide removes magnesium induced inhibition and enables excitatory inflammatory proteins such as glutamate and aspartate to activate specialized spinal cord NMDA receptors and increase magnification of the nerve traffic and pain stimuli.

Axial and radicular back pain is symptoms of injury that result in a cascade of inflammatory mediators. Local anesthetic agents stabilize nerve membrane and decrease pain by reducing the rate of discharge of sensory nerve fibers and decreasing neurogenic inflammation [16].

Current procedural injections for back, neck and radicular pain focus on structures that are visible with imaging, skeletal system (bones, joints, intervertebral discs) and central nervous system (brain and spinal cord) components while completely ignoring the largest organ in the spine and most often the initial site of injury, which are the paraspinal muscles–cervical, thoracic and lumbar. The most common pain procedures are essentially blind techniques as pain triggers are targeted based upon structural pathology. Unfortunately there is poor correlation between structural pathology and the presence of back [17,18] or neck pain [19].

Myofascial tissues constitute a pain generator that is not addressed in current interventional pain procedures. Randomized controlled studies of current procedures have yielded conflicting results. In a study by Dilke et al., [20] patients who received epidural corticosteroids experienced less pain than controls, needed surgery less often and returned back to work sooner. However and on the contrary, Snoek et al., [21] showed that epidural steroid injection was no more effective than a placebo injection in relieving chronic symptoms due to lumbar disc herniation. In 2008, Staal et al. [22] reported on a systematic review of the literature conducted with a focus on randomized, controlled trials. Based on their analyses, the authors made the conclusion that there is moderate evidence that epidural corticosteroid injections are no more effective than placebo injections for pain relief.

Other procedures range from transforaminal injections, radiofrequency thermal lesioning of the medial branch nerves, radiofrequency cryolesioning of the medial branch nerves, intradiscal electrothermy (thermal burn to the intervertebral disc), and spinal cord stimulation. Recent randomized control trials have also questioned the effectiveness of these more invasive procedures. A multicenter, randomized, double-blind, sham treatment controlled trial was performed to determine the efficacy of radiofrequency lumbar facet joint denervation. In both groups, there was significant improvement in the Visual Analog Scale (VAS) [23]. In the Mint study, three randomized clinical trials were conducted on the effectiveness of minimal interventional treatments for participants with chronic low back pain. Radiofrequency denervation combined with a standardized exercise program resulted in either no improvement or no clinically important improvement in chronic low back pain compared with a standardized exercise program alone. In another randomized control trial, assessing the efficacy of Radiofrequency (RF) denervation of the cervical facet joints in chronic cervical facet joint pain the authors stated that they did not observe significant differences between RF denervation combined with injection of local anesthesia compared with local anesthesia at 6 months follow-up. The need for pain medication did not differ significantly between groups [24]. In another randomized controlled trial, in patients with cervicogenic headache the authors did not find evidence that radiofrequency treatment of cervical facet joints was a better treatment than the infiltration of the greater occipital nerve [25].

In one study the author stated that the evidence is poor for cervical transforaminal epidural injections. Complications with cervical interlaminar epidural injections are rare, but more commonly occur with transforaminal epidural injections. These can be fatal and include vertebral artery injury, systemic allergic reactions to radio contrast agents, transient cortical blindness and brain injury [26], stroke, paraplegia, quadriplegia, spinal cord and cerebella infarction [27,28].

These trials raise the issue of questionable benefits, compared to simpler procedures such as our myofascial nerve blockade.

As stated in the NIH, HEAL Initiative Fund Opportunity [29], the field of musculoskeletal pain has largely focused on the skeletal system (bones, joints, intervertebral discs) and central nervous system (brain and spinal cord) components. The contribution of myofascial tissues, especially fascia, and the interactions of fascia, muscles, and peripheral nerves are understudied and remain mostly unknown. Pain originating from muscles and fascia is likely an important component of many severe and chronic pain conditions. The perimuscular fascia is richly innervated with small-diameter fibers whose receptive fields increase in the presence of inflammation. Thus myofascial tissues play a significant role as pain generators.

The NIH noted that for many years, structural imaging was the main tool to guide treatment decisions, including surgery. Meanwhile, imaging and other objective measurements of “soft” tissues including muscles, and connective tissues or “fasciae,” were not even considered as musculoskeletal pain biomarker candidates. Thus NIH has called for development of biomarkers of myofascial tissues for effective pain management regimens.

In the last seven years, we have replaced epidural and facet joint/nerve injections with a myofascial nerve block. We routinely perform myofascial nerve injections, with or without ultrasound guidance to relieve pain. We had observed during spinal procedures that a clear solution of Lidocaine 2%, injected from a 30G 5/8 inch (15.6 mm) needle, using a 3 ml syringe, and inserted just lateral to the spinous process, into the paraspinal muscles, traveled a distance of 4cm to 6 cm [4]. This distance was sufficient to travel through the erector spinae muscles down to the vertebral lamina and posterior spinal structures and within minutes produce anesthetic block to relieve radicular pain from the nerve roots [4].

**Figure 1:**
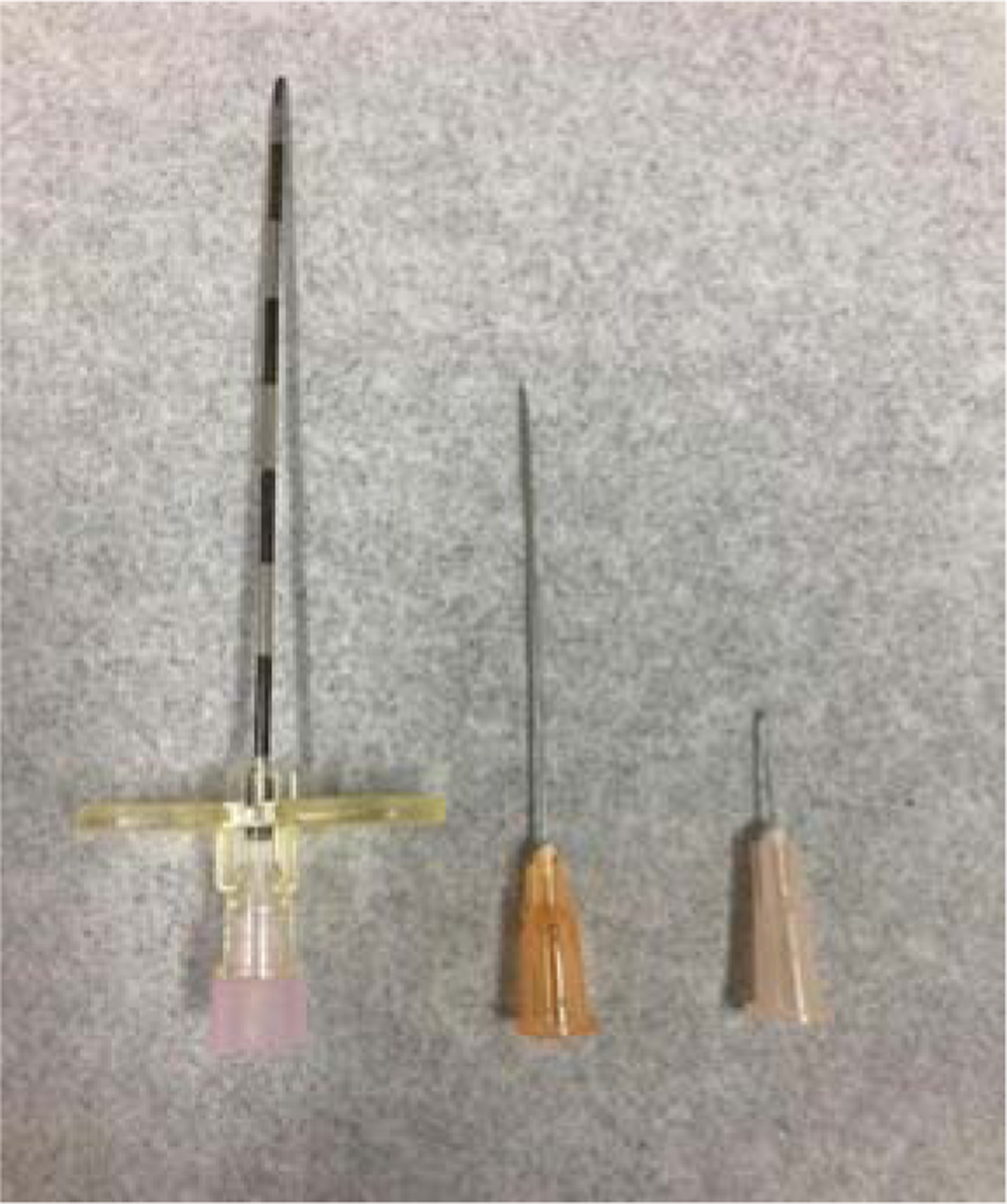
Comparison of a 30 G needle with an epidural and spinal needle.

**Figure 2:**
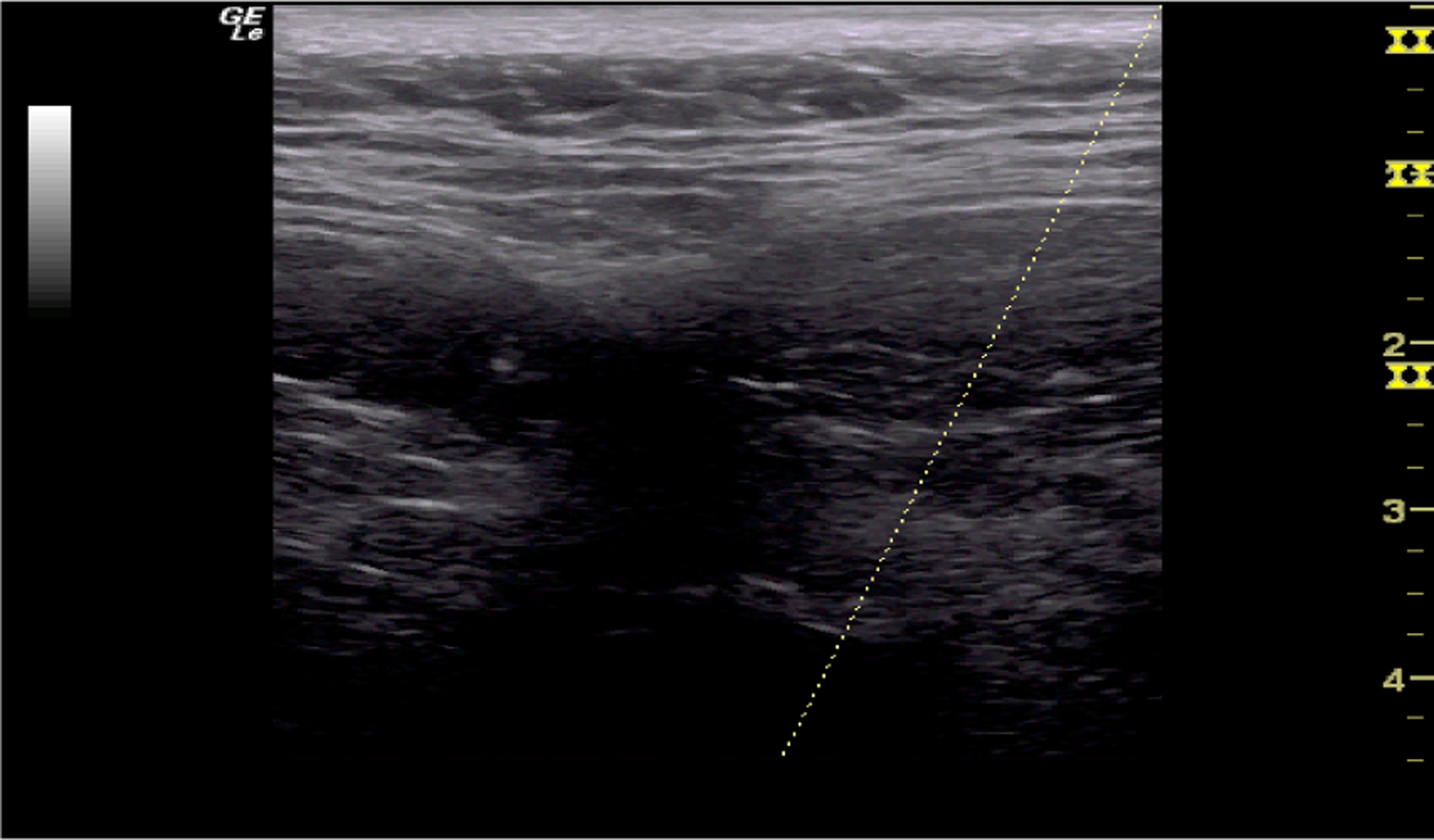
Ultrasound screen showing the depth reached by medication in tissue using a 30 G needle during a trigger point injection of the lumbar paraspinal muscle. From the screen of the ultrasound, the medication travels 4 cm to 6 cm (40 mm to 60 mm) into the muscle.

**Figure 3:**
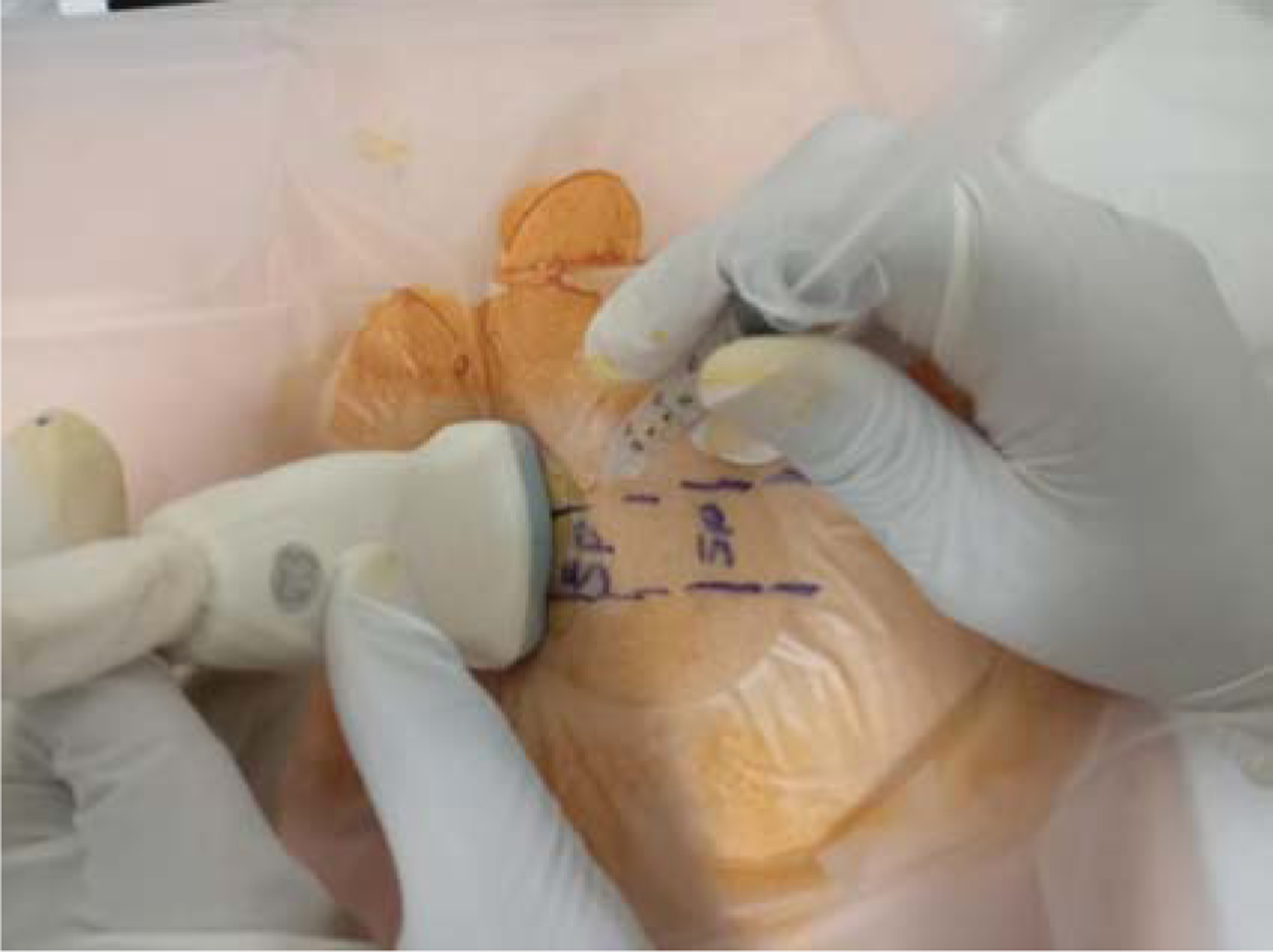
Myofascial Injection technique with ultrasound guidance. (SP: Spinous Process).

**Figure 4:**
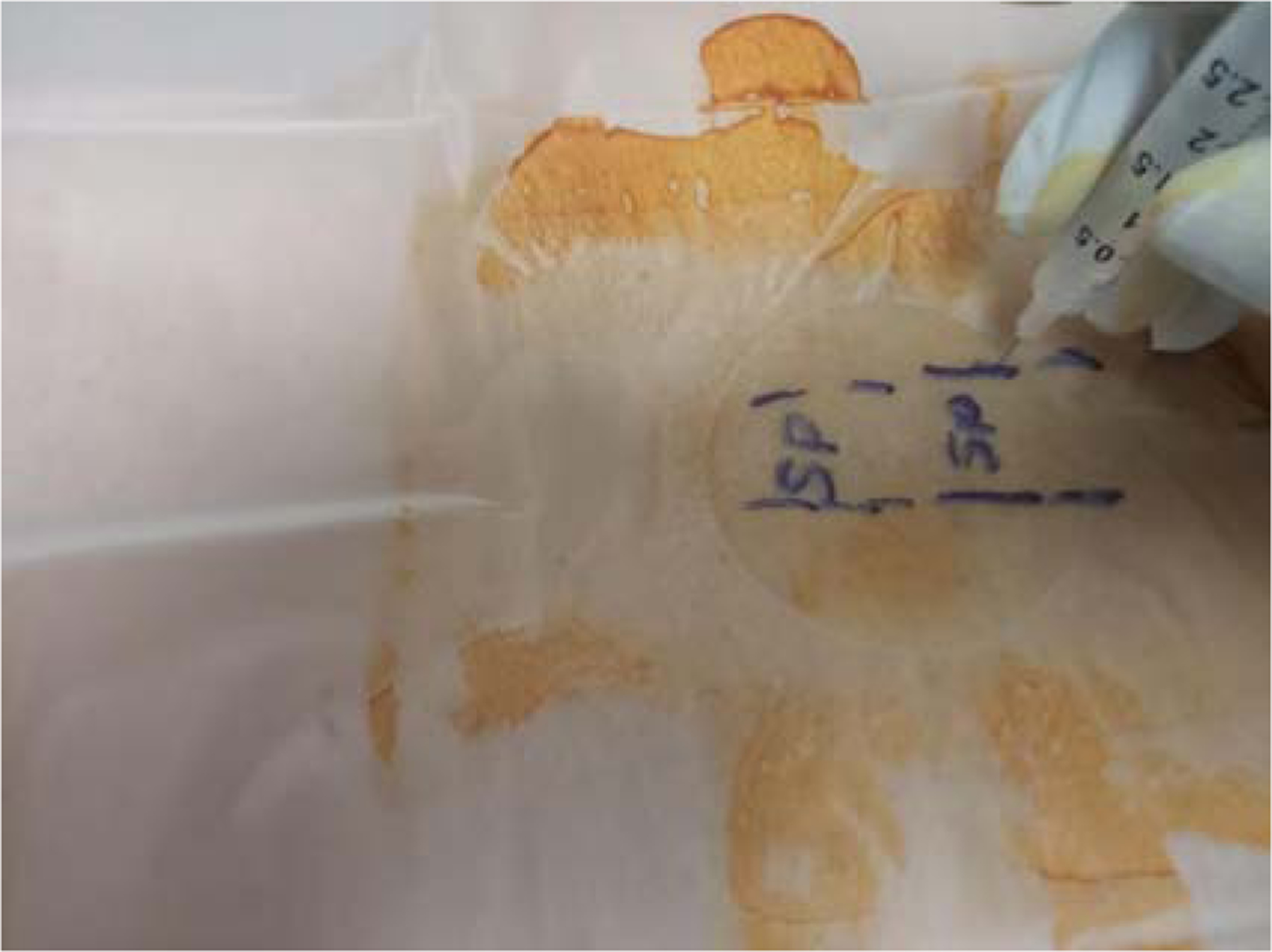
Myofascial Injection technique-Showing 5/8 in length of the 30G needle.

**Figure 5:**
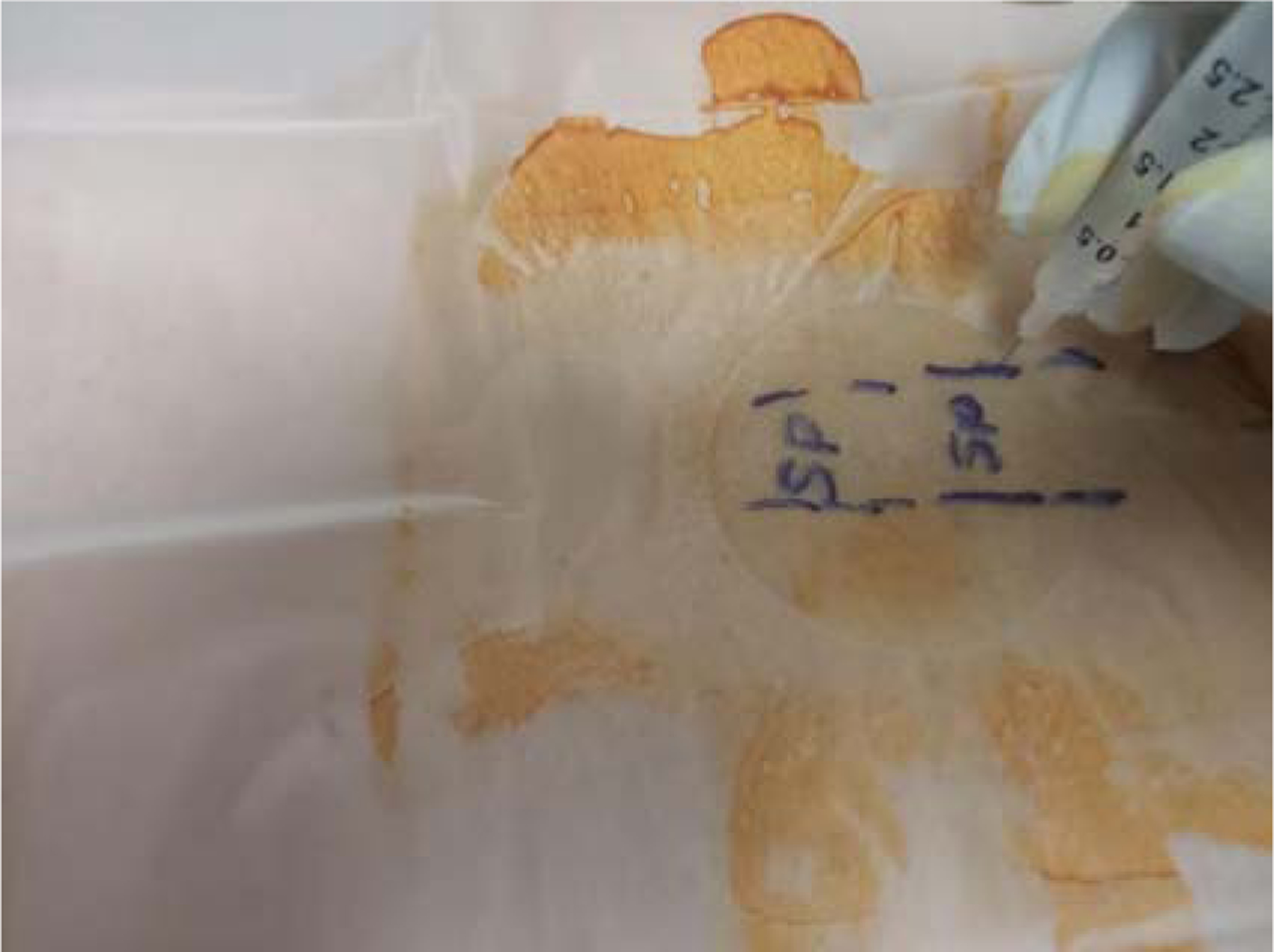
Myofascial Injection technique, without ultrasound guidance.

**Figure 6:**
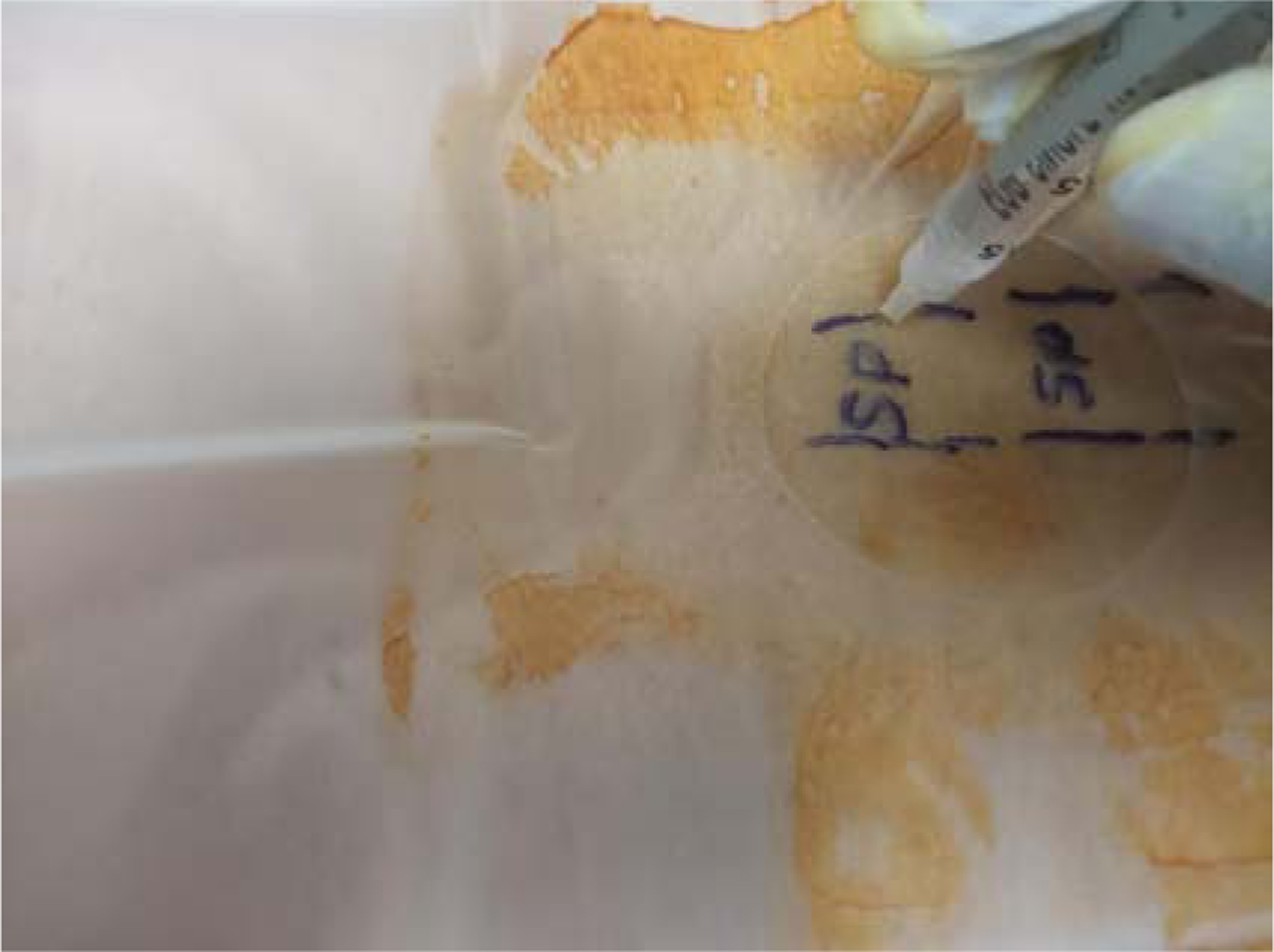
Myofascial Injection technique, needle halfway inserted.

In the last two years, the advent of erector spinae plane blocks that are essentially myofascial blocks but utilizing longer needles inserted to the target point, with large volumes of dilute local anesthetic has validated our original but simpler myofascial nerve block (Figures 1–6 and Video 1).

## Discussion

When a 30 gauge needle is attached to a 3 ml syringe, the distance of travel of medication from the syringe and needle into tissue will be greater, because there is increased velocity through the smaller needle and hence greater penetration of medication into the tissues.

**Figure 7:**
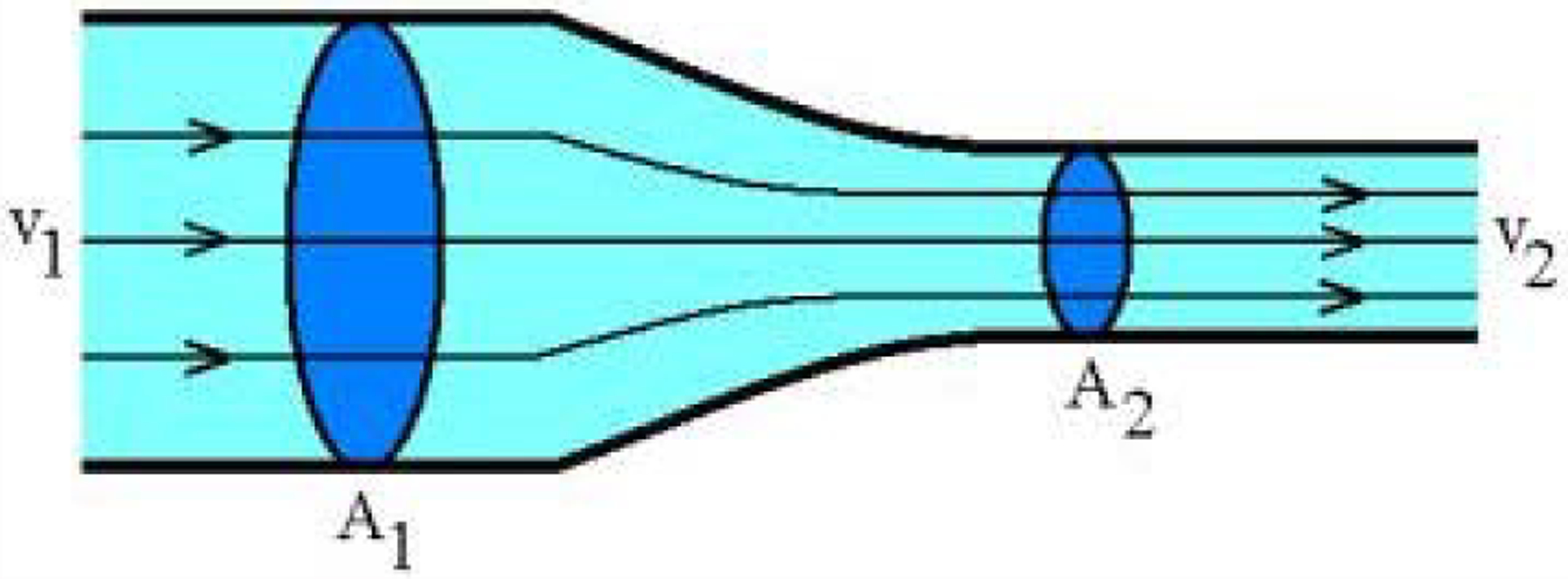
The pipe constricting from area A1 to area A2.

Utilizing the equation of continuity, we can analyze what happens to the fluid if the size of the tubing through which it flows, changes. Figure 7 shows the pipe constricting from area A_1_ to area A_2_. Since no fluid can leave through the walls, the mass crossing each section of the tube per unit time must be the same. Therefore the velocity of fluid through the smaller area is faster than the velocity of the fluid through the larger area. This phenomenon can be explained and quantified by examining the flow rate of mass through the tubing. The equation of continuity states that, in any steady state process, the rate at which mass enters a system is equal to the rate at which mass leaves the system:

Flow rate through *A*_*1*_ = Flow rate through *A*_*2*_

d_1_A_1_v_1_ = d_2_A_2_v_2_

Therefore,

*dAv* = Constant

This equation expresses the law of conservation of mass in fluid dynamics.

If fluid is incompressible, then the density is constant (d_1_ = d_2_), Then,

A_1_v_1_=A_2_v_2_

Where

A1=Area in tube 1

v1=Velocity in tube 1

A2=Area in tube 2

v2=Velocity in tube 2

d=density of the fluid

For our purpose, A1v1 will be a syringe and A2v2 the hypodermic needle. When a higher gauge (smaller) needle is used, with a 3 ml syringe, the distance of travel of medication from the syringe and needle into tissue will be greater, because there is increased velocity through the smaller needle and hence greater penetration of medication into the tissues.

### Advantages

Clear fluid medications can be injected to traverse and provide a therapeutic effect at a distance to the needle point e.g. paraspinal muscles and lamina.

### Limitations

This targeted structure must be within 6 cm distance and not obstructed by bone e.g. a hip or knee joint.

## Myofascial Injection Technique

With the Myofascial Injection Technique for Spinal Pain, the spine is palpated to locate the most painful sites. A short 30G 5/8 inch needle is inserted adjacent to the spinal process or vertebra at the level of those sites. The soft tissue is compressed and the needle is advanced just past the subcutaneous tissue into the muscle layer, and 1 ml to 2 ml of local anesthetic solution (preferably 2% Lidocaine for most sites) is injected in each site. If performed under ultrasound guidance the solution will be seen to spread 4 cm to 6 cm deep into the paraspinal muscle, down to the lamina and posterior spinal structures. The injection is repeated at the 2 to 4 most painful sites, keeping in mind not to exceed the toxic dose of the local anesthetic (3 mg/kg). The number of sites injected is limited by the volume and concentration of local anesthetic injected so as not to exceed the toxic dose of anesthetic.

With the myofascial injection, the soft tissue is compressed and the needle is inserted close to its hub (5/8 in), the medication is injected to reach the target site. In most instances, it is no longer necessary to use longer and bigger gauge needles for epidural injections for treatment of axial pain and radiculopathy. With a myofascial injection, the medication can be pushed under pressure through the erector spinae muscle to block the posterior spinal structures, using a smaller shorter needle. Use of such a short small bore needle such as the BD 30G 5/8 inch needle will make myofascial injections much safer as there is significantly decreased risk of needle trauma. This technique is also useful in patients who are anti-coagulated. Compression of the skin and subcutaneous fatty tissue reduces the distance of travel required for the medication to penetrate through the paraspinal muscle to the posterior spinal structures.

## Distances

Clear solution of medication injected under pressure from a 30G 5/8 inch (15.6 mm) needles travel a distance of 4 cm to 6 cm.

**Figure 8:**
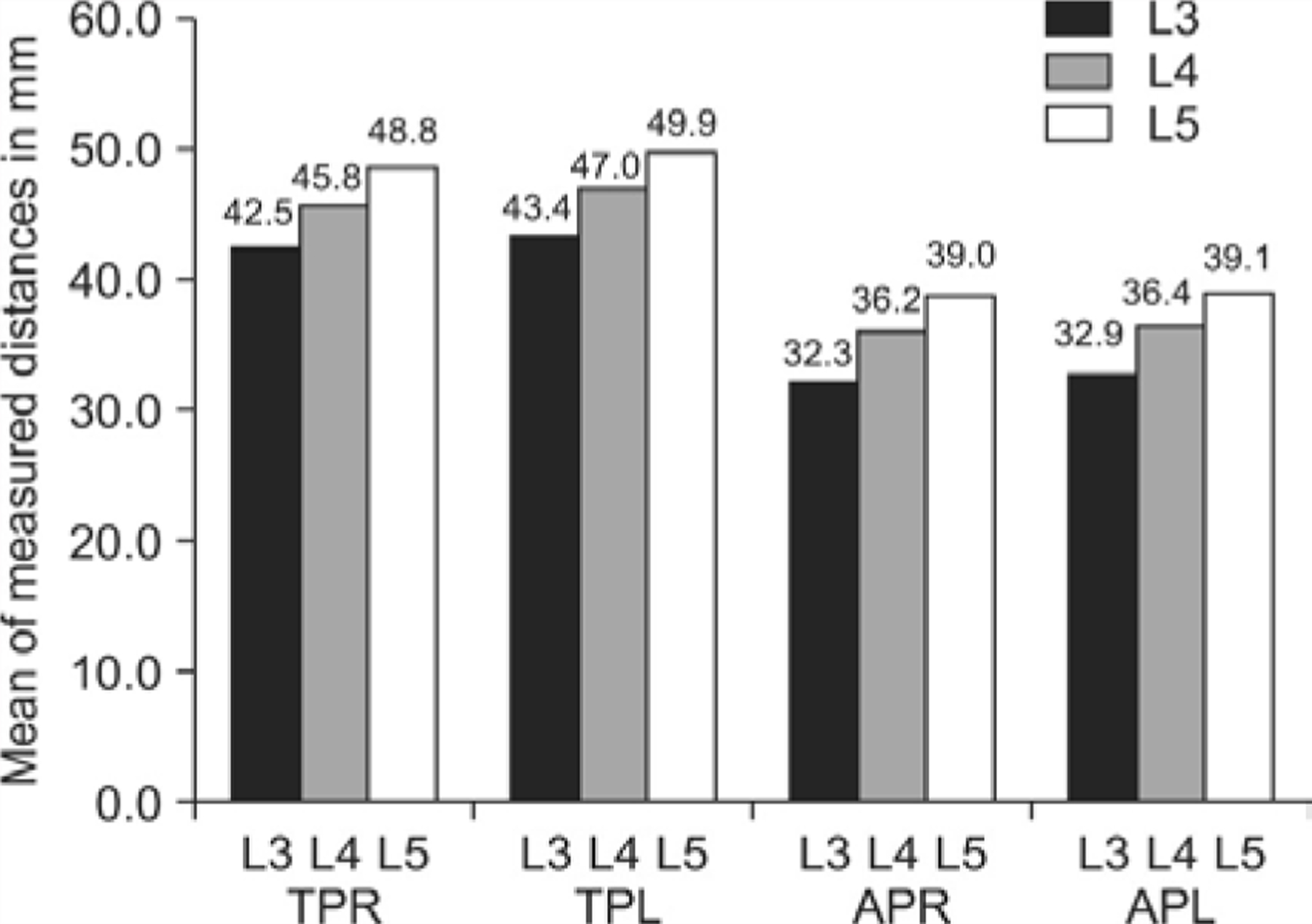
Mean distances (mm) between skin and transverse processes (TPR &TPL) and skin and articular processes (APR & APL) by vertebral level (TPR: skin to transverse process at the right side, TPL: skin to transverse process at the left side, APR: skin to articular process at the right side. APL: skin to articular process at the left side). It can therefore be seen that by compressing the soft tissue and shortening the distance, with our technique for procedural injections utilizing a short small bore needle such as the BD 30G 5/8 inch needle, medication can be delivered at a distance, under pressure, to travel to block pain generators including peripheral nerves in the muscle, fascia, as well as in the posterior spinal structures in including the medial branch nerves, facet joints and lumbar nerve roots. This is the first procedural technique that can block multiple pain generators in just one injection.

In the lumbar spine, the distance from the skin to the ligamentum flavum is 3 cm to 8 cm [30–32]. In the upper thoracic spine the distance from skin to the lamina with a paramedian approach, 1 cm from the midline, is 4.2 cm, middle thoracic spine is 3.7 cm, lower thoracic spine is 3.6 cm and lumbar spine is 4.0 cm. The average distance from skin to the epidural space with a paramedian approach, 1 cm from the midline, in the upper thoracic spine is 5.6 cm, middle thoracic spine is 5.2 cm, lower thoracic spine is 4.4 cm and lumbar spine is 4.7 cm [33]. The mean distance from the skin to the transverse processes and facet joint articular processes, ranges from 3.2 cm to 5 cm, as measured in a study utilizing a 3 MHz to 5 MHz ultrasound probe see Figure 8 [34].

We have had a variable duration of pain relief from one week to one year and similar to the epidural steroid and facet nerve injections that we previously performed. There have been no complications with this technique, in more than 500 procedures.

## Conclusion

The Myofascial nerve injection is a simple intervention technique that can be performed from the medical clinic to the battlefield to quickly relieve inflammation, treat acute pain and prevent chronic pain.

It is the first procedure that targets the myofascial tissues, peripheral innervations, posterior spinal structures and spinal nerve roots at the same time.

Due to the small size and length of the needle, this technique can be performed not just by pain specialist but by a primary care physician, physician assistant, nurse practitioner or any medical service provider trained to do intramuscular injections.

The therapeutic and anti-inflammatory effect of the myofascial nerve injection is from the neuro-modulatory activity local anesthetic (preferably 2% Lidocaine for most injection sites). The myofascial injection procedure using the Sota Omoigui Short Needle Technique may be combined with an anti-inflammatory regimen that may be administered by the parenteral route.

## Figures and Tables

**Video 1: Video of ultrasound during myofascial injection procedure.** 

## References

[1] Ter Meulen BC, Weinstein H, Ostelo R, Koehler PJ. The epidural treatment of sciatica: Its origin and evolution. Eur Neurol 2016;75(1–2):58–64. 26820578 10.1159/000443729

[2] Sicard MA. Les injections médicamenteuses extradurales par voie sacrococcygienne. C R Seances Soc Biol Fil. 1901;53:396.

[3] Cathelin F Une nouvelle voie d’injection rachidienne: Méthode des injections épidurales par le procédé du canal sacre - applications à l’homme. Compt Rend Soc de Biol 1901.

[4] Omoigui S, Ogbeiche C, Fadare A. Reinventing IM and procedural injections: The Sota Omoigui short needle technique. Practical Pain Management 2016:28–34.

[5] Omoigui S Merging physics with anesthesia presented at the International Anesthesia Research Society Conference, May 16th, 2019, Montreal, Quebec, Canada.

[6] Forero M, Adhikary SD, Lopez H, Tsui C, Chin KJ. The erector spinae plane block: A novel analgesic technique in thoracic neuropathic pain. Reg Anesth Pain Med 2016;41(5):621–7. 27501016 10.1097/AAP.0000000000000451

[7] Ueshima H, Hiroshi O. Spread of local anesthetic solution in the erector spinae plane block. J Clin Anesth 2018;45:23. 29258057 10.1016/j.jclinane.2017.12.007

[8] Ivanusic J, Konishi Y, Barrington MJ. A cadaveric study investigating the mechanism of action of erector spinae blockade. Reg Anesth Pain Med 2018;43(6):567–71. 29746445 10.1097/AAP.0000000000000789

[9] Tsui BC, Mohler D, Caruso TJ, Horn JL. Cervical erector spinae plane block catheter using a thoracic approach: An alternative to brachial plexus blockade for forequarter amputation. Can J Anaesth 2019;66(1):119–20. 29868941 10.1007/s12630-018-1170-7

[10] Jadon A, Jain P, Sinha N. The erector spinae plane block for postoperative analgesia in abdominoplasty - A case report. BAOJ Anesthesia 2017;1(1):001.

[11] Chin KJ, El-Boghdadly K. Mechanisms of action of the Erector Spinae Plane (ESP) block: A narrative review. Can J Anaesth 2021;68(3):387–408. 33403545 10.1007/s12630-020-01875-2

[12] Omoigui S The biochemical origin of pain: How a new law and new drugs have led to a medical breakthrough in the treatment of persistent pain. State of the Art Technology Publishers 2002.

[13] Dieleman JL, Baral R, Birger M, Bui AL, Bulchis A, Chapin A, US spending on personal health care and public health, 1996–2013. JAMA 2016;316(24):2627–46. 28027366 10.1001/jama.2016.16885 PMC5551483

[14] Omoigui S The biochemical origin of pain--proposing a new law of pain: The origin of all pain is inflammation and the inflammatory response. Part 1 of 3--a unifying law of pain. Med Hypotheses 2007;69(1):70–82. 17240081 10.1016/j.mehy.2006.11.028 PMC2766416

[15] Omoigui S The biochemical origin of pain: The origin of all pain is inflammation and the inflammatory response. Part 2 of 3-inflammatory profile of pain syndromes. Med Hypotheses 2007;69(6):1169–78. 17728071 10.1016/j.mehy.2007.06.033 PMC2771434

[16] Waxman SG. The molecular pathophysiology of pain: Abnormal expression of sodium channel genes and its contributions to hyperexcitability in primary sensory neurons. Pain 1999;6:S133–S140. 10.1016/S0304-3959(99)00147-5 10491982

[17] Boden SD, Davis DO, Dina TS, Patronas NJ, Wiesel SW. Abnormal magnetic-resonance scans of the lumbar spine in asymptomatic subjects. A prospective investigation. J Bone Joint Surg Am 1990;72(3):403–8. 2312537

[18] Jensen MC, Brant-Zawadzki MN, Obuchowski N, Modic MT, Malkasian D, Ross JS. Magnetic resonance imaging of the lumbar spine in people without back pain. N Engl J Med 1994;331(2):69–73. 8208267 10.1056/NEJM199407143310201

[19] Van der Donk J, Schouten JS, Passchier J, van Romunde LK, Valkenburg HA. The associations of neck pain with radiological abnormalities of the cervical spine and personality traits in a general population. J Rheumatol 1991;18(12):1884–9. 1795327

[20] Dilke TF, Burry HC, Grahame R. Extradural corticosteroid injection in management of lumbar nerve root compression. Br Med J 1973;2(5867):635–7. 4577015 10.1136/bmj.2.5867.635 PMC1589689

[21] Snoek W, Weber H, Jorgensen B. Double blind evaluation of extradural methyl prednisolone for herniated lumbar discs. Acta Orthop Scand 1977;48(6):635–41. 343479 10.3109/17453677708994810

[22] Staal JB, de BR, de Vet HC, Hildebrandt J, Nelemans P. Injection therapy for subacute and chronic low‐back pain. Cochrane Database Syst Rev 2008;2008(3):CD001824. 18646078 10.1002/14651858.CD001824.pub3 PMC7096223

[23] Van Wijk RM, Geurts JW, Wynne HJ, Hammink E, Buskens E, Lousberg R, Radiofrequency denervation of lumbar facet joints in the treatment of chronic low back pain: A randomized, double-blind, sham lesion-controlled trial. Clin J Pain 2005;21(4):335–44. 15951652 10.1097/01.ajp.0000120792.69705.c9

[24] van Eerd M, de Meij N, Kessels A, Patijn J, Weber W, Wintraecken V, Efficacy and long-term effect of radiofrequency denervation in patients with clinically diagnosed cervical facet joint pain: A double-blind randomized controlled trial. Spine (Phila Pa 1976) 2021;46(5):285–93. 33534439 10.1097/BRS.0000000000003799

[25] Haspeslagh SR, Van Suijlekom HA, Lamé IE, Kessels AG, van Kleef M, Weber WE. Randomised controlled trial of cervical radiofrequency lesions as a treatment for cervicogenic headache [ISRCTN07444684]. BMC Anesthesiol 2006;6:1. 16483374 10.1186/1471-2253-6-1 PMC1403750

[26] McMillan MR, Crumpton C. Cortical blindness and neurologic injury complicating cervical transforaminal injection for cervical radiculopathy. Anesthesiology 2003;99(2):509–11. 12883429 10.1097/00000542-200308000-00038

[27] Tiso RL, Cutler T, Catania JA, Whalen K. Adverse central nervous system sequelae after selective transforaminal block: The role of corticosteroids. Spine J 2004;4(4):468–74. 15246308 10.1016/j.spinee.2003.10.007

[28] Manchikanti L, Falco FJ, Diwan S, Hirsch JA, Smith HS. Cervical radicular pain: The role of interlaminar and transforaminal epidural injections. Curr Pain Headache Rep 2014;18(1):389. 24338702 10.1007/s11916-013-0389-9

[29] NIH Heal Initiative. Department of Health and Human Services

[30] Joseph P Geophysical fluid dynamics Springer. 1987:10–3.

[31] Bloomfield Louis. How Things Work: The physics of everyday life 3^rd^ Ed. John Wiley and Sons; 2006. p. 153. ISBN 0-471-46886-X.

[32] Neuraxial Blockade Anatomy and Landmarks

[33] Ogura A, Inoue T, Wajima Z, Yoshikawa T, Imanaga K. Skin to vertebral lamina distance as a principal landmark for the epidural puncture using the paramedian approach. Eur J Anaesthesiol 2000;17:96.

[34] Gharaei H, Imani F, Solaymani-Dodaran M. Survey of sonoanatomic distances for lumbar medial branch nerve blocks in healthy volunteers. Korean J Pain 2014;27(2):133–8. 24748941 10.3344/kjp.2014.27.2.133 PMC3990821

